# Novel Polyvinyl Alcohol/Starch Electrospun Fibers as a Strategy to Disperse Cellulose Nanocrystals into Poly(lactic acid)

**DOI:** 10.3390/polym9040117

**Published:** 2017-04-07

**Authors:** Carol López de Dicastillo, Karina Roa, Luan Garrido, Alejandro Pereira, Maria Jose Galotto

**Affiliations:** 1 Food Packaging Laboratory (Laben), Department of Science and Food Technology, Faculty of Technology, Center for the Development of Nanoscience and Nanotechnology (CEDENNA), Universidad de Santiago de Chile (USACH), 9170201 Santiago, Chile; karina.roa@usach.cl (K.R.); luan.garrido.a@gmail.com (L.G.); maria.galotto@usach.cl (M.J.G.); 2 Faculty of Physics, Center for the Development of Nanoscience and Nanotechnology (CEDENNA), Universidad de Santiago de Chile (USACH), 9170201 Santiago, Chile; apereira82@gmail.com

**Keywords:** electrospinning, polyvinyl alcohol, starch, poly(acid lactic), cellulose nanocrystals

## Abstract

In this work, electrospun fibers of polyvinyl alcohol (PV) and starch (ST) were obtained to improve dispersion of cellulose nanocrystals (CNC) within a poly(lactic acid) (PLA) matrix with the aim of enhancing mechanical and barrier properties. The development and characterization of electrospun fibers with and without CNC, followed by their incorporation in PLA at three concentrations (0.5%, 1% and 3% with respect to CNC) were investigated. Morphological, structural, thermal, mechanical and barrier properties of these nanocomposites were studied. The purpose of this study was not only to compare the properties of PLA nanocomposites with CNC embedded into electrospun fibers and nanocomposites with freeze-dried CNC, but also to study the effect of electrospinning process and the incorporation of CNC on the PV and starch properties. SEM micrographs confirmed the homogenous dispersion of fibers through PLA matrix. X-ray analysis revealed that the electrospinning process decreased the crystallinity of PV and starch. The presence of CNC enhanced the thermal stability of electrospun fibers. Electrospun fibers showed an interesting nucleating effect since crystallinity of PLA was strongly increased. Nanocomposites with electrospun fibers containing CNC presented slightly higher flexibility and ductility without decreasing barrier properties.

## 1. Introduction

Over the last several years, the efforts to improve the properties of biodegradable materials have increased due to the effects that residues of conventional materials have on the environment [1]. The introduction of nanotechnology in the development of new materials has opened up a great number of possibilities. Adding nanofillers has presented an interesting way to extend and to improve some aspects, principally mechanical and barrier properties [2]. A nanocomposite is a multiphase composite where at least one of the phases presents nanoscale dimension. There are many nanofillers (three-dimensional spherical and polyhedral, two-dimensional nanofibers or one-dimensional sheet-like nanoparticles) that have been studied, but during the last decade, “cellulose nanocrystals” (CNC) have attracted significant interest in order to produce fully renewable and biodegradable nanocomposites. CNC is a natural nanofiller obtained from cellulose, a fibrous, hard and water-insoluble substance that plays an essential role in maintaining the structure of plant cell walls. The multiple networks between cellulose chains through hydrogen bonding constitute cellulosic fibrils that have highly ordered (crystalline) and unordered (amorphous) regions. Amorphous regions can be selectively hydrolyzed through acid hydrolysis, obtaining nanosized crystalline regions called nanocellulose or “cellulose nanocrystals” [3,4]. As compared to inorganic reinforcing fillers, CNC have many additional advantages including wide availability of sources, low-energy consumption, ease of recycling by combustion, high aspect ratio and good mechanical properties [5,6]. Several studies have associated the incorporation of CNC with improvements in dynamic mechanical thermal properties, tensile strength, toughness and elongation at break [7,8,9,10]. Nevertheless, one of the main difficulties associated with the use of CNC as reinforcing agents is its high hydrophilicity and strong hydrogen bond interactions, which make it difficult to disperse in hydrophobic media, including most widely researched thermoplastic biopolymers, such as poly(lactic acid), PLA. Although PLA is one of the most popular bio-based plastics and finds wide industrial use nowadays, this biopolyester still presents some drawbacks as low thermal resistance, excessive brittleness and high oxygen permeability. These issues are mainly due to their low crystallizing nature, and different processes have been employed to improve these properties [11]. Thus, some strategies have been devised in order to improve dispersion of CNC into polymer matrices, such as grafting and chemical surface modification of CNC, masterbatch in situ polymerization, the use of surfactants and partial silylation [12,13,14,15,16]. Nevertheless, most of these modifications are complicated processes, and results have demonstrated that the modified CNC have less reinforcing effects. In this work, the alternative proposed was the incorporation of CNC into poly(acid lactic), PLA, by means of electrospinning with polyvinyl alcohol, PV, and starch, ST. The principal aim of this work was the enhancement of mechanical and barrier properties of PLA through the development and the study of these electrospun fibers as an efficient strategy to successfully disperse CNC into this hydrophobic biopolymer and compare the properties of these materials with PLA nanocomposites with freeze-dried CNC. 

Electrospinning is an economical, simple and versatile technique to deposit polymer fibers with dimensions from micrometers down to nanometers onto a target using an electric field to regulate the ejection of the polymeric fluid jet from the syringe [17]. Polyvinyl alcohol (PV) and starch (ST) were selected due to their hydrophilic and water soluble nature. PV is a semi-crystalline polymer with good chemical and thermal stability. Electrospun fibers produced from PV and mixtures with other polymers have been widely studied over the past few years because of its non-toxic and biodegradability [18,19]. On the other hand, starch is among the most abundant and inexpensive biopolymer, since it is found in plant issues, such as leaves, stems, seeds and roots. It is composed of repeating glucose monomers, and is found in its linear form as amylose, and in a branched form as amylopectin. However, pure starch lacks the strength, water resistibility, thermal stability and processability. Several attempts have been made to fabricate starch fibers, such as processing only amylose fibers, utilizing modified starches, changing solvents, or including plasticizers, crosslinker resin or other polymers [20,21,22]. The combination PV-ST is ideal because PV is usually modified with other polymers, such as starch, to improve its biodegradability and performance. Additionally, few works have centered their attention on the electrospinning of PV and ST [23,24]. Several works have used this technique with the purpose to improve physical properties of different polymers. Narayanan et al. studies have shown that the reinforcement of poly(ε-caprolactone) with cyclodextrin resulted in improvements in mechanical and thermal properties [25,26,27]. Martinez-Sanz et al. [28] have already incorporated bacterial cellulose nanocrystals into PLA through PLA electrospun fibers resulting on materials with higher values of tensile strength and elastic modulus, but lower elongation and barrier properties. In this work, the advantage by using hydropillic polymers, PV and starch, is to avoid the need of freeze-drying the CNC solution, which is one of the longest and most energetically costly process in the CNC obtaining procedure. In addition to compare the properties of PLA nanocomposites with CNC embedded into PV/ST electrospun nanofibers and nanocomposites with direct addition of freeze-dried CNC, the study of the effect of electrospinning process and the incorporation of CNC in the PV and starch properties was also investigated.

## 2. Materials and Methods

### 2.1. Materials and Nanoreinforcements

#### 2.1.1. Polymers and Chemicals

Poly(lactic acid) (PLA), 2003D (specific gravity ¼ 1.24; MFR g/10 min (210 °C, 2.16 kg)) was purchased from Natureworks^®^ Co. (Minnetonka, MN, USA). Gohsenol type AH-17 polyvinyl alcohol (PV) (saponification degree 97%–98.5% and viscosity 25–30 mPa s) was obtained from the Nippon Synthetic Chemical Co. (Osaka, Japan). Starch (ST), cellulose fibers (CF) (powder 80–145 µm), and polyethylene glycol (PEG) were supplied by Sigma Aldrich (Santiago, Chile). Chloroform and sulfuric acid 95%–97% were supplied by Merck (Santiago, Chile). Low-flow PES (polyethersulfone) 170 dialysis membranes (35 µm thickness, 20.000 Da pore size) were purchased from the Nipro Medical Corporation (Santiago, Chile).

#### 2.1.2. Preparation of Cellulose Nanocrystal Solution

Ten grams of cellulose fibers (CF) was mixed with 50 mL of deionized water and put in an ice bath and stirred while 50 mL of concentrated sulfuric acid were added dropwise until the solution achieved 9 M concentration. The suspension was then heated at 45 °C and stirred for 120 min, followed by the addition of water to stop the hydrolysis. The resulting mixture was centrifuged at 4000 rpm for 20 min, and the clear supernatant containing acid residues and amorphous regions of the cellulose fiber was removed. Subsequently, successive washings were performed adding 50 mL of distilled water and the tubes were shaken again and centrifuged at 4000 rpm for 12 min. This operation was repeated until the supernatant was a turbid suspension containing the CNC [29,30]. The suspension obtained was dialyzed until the washing water maintained at constant pH. A known volume of the previous CNC suspension was freeze-dried to calculate the concentration of CNC and to obtain dry CNC to cast CNC nanocomposites used as control nanocomposites.

#### 2.1.3. Electrospun PV/Starch Nanofibers

CNC solution (CNC-A) obtained from dialysis was concentrated through evaporation until a final concentration of 2% (*w*/*v*) (CNC-B) with the purpose to achieve a high incorporation degree of CNC into the electrospun fibers and lowest influence of PV/starch polymers in the blends with PLA. Furthermore, 1.6 g PV and 0.6 g starch were added to 20 mL of CNC-B solution and stirred at 90 °C until polymers were dissolved. In order to study the incorporation of CNC into the fiber, PV/starch solution at the same concentration without CNC was also prepared to be electrospun. Solutions were transferred to 5 mL plastic syringes and connected through a PTFE (polytetrafluoroethylene) tube to an 18-gauge blunt stainless steel needle charged by a high voltage power supply with a range of 0–30 kV. The collector plate was fixed at a working distance of 8.5 cm below the needle tip and connected to the grounded counter electrode of the power supply. A voltage of approximately 15 kV and a flow rate of 1.5 mL/h were used. CNC containing PV/starch fibers were named “(PVST/CNC)_f_“ and PV/starch nanofibers “(PVST)_f_”.

### 2.2. PLA Nanocomposite Preparation

PLA based films were obtained by solution-extension-evaporation process (“casting”). Electrospun fibers (PVST/CNC)_f_ were mixed with PLA solution in chloroform in order to obtain blends with a final concentration of 0.5, 1 and 3 wt % CNC respect PLA weight (film codes: “0.5PLA(PVST/CNC)_f_, 1PLA(PVST/CNC)_f_ and 3PLA(PVST/CNC)_f_”, respectively). PEG was added at 5% (*w*/*w* polymer) for all formulations to facilitate the casting process. Two different control nanocomposite films were also casted: (i) with electrospun fibers (PVST)_f_ owing to clarify if the differences on material properties were due to the fibers or to the presence of CNC: “0.5PLA(PVST)_f_, 1PLA(PVST)_f_ and 3PLA(PVST)_f_”; and (ii) with lyophilized CNC: “0.5PLACNC, 1PLACNC and 3PLACNC”) in order to verify the hypothetical improvement expected due to the encapsulation of nanocellulose into these biodegradable electrospun fibers. Table 1 shows the content of each component (in %) to develop every film in order to reach CNC concentrations of 0.5%, 1% and 3%, and the corresponding control films. PLA blank (only with PEG) was also casted and named “PLA”.

### 2.3. Scanning Electronic Microscopy (SEM) Analysis

The morphology of the electrospun fibers (PVST/CNC)_f_ and (PVST)_f_ and the nanocomposites were studied using a scanning electron microscope (SEM) JSM-5410 Jeol (Tokyo, Japan) with accelerating voltage at 10 kV. Films were fracturated using a Tensile Tester because it was not possible to obtain the samples through cryo-fracture. Then, samples were coated with gold palladium using a Sputtering System Hummer 6.2., and SEM micrographs of the surface and the cross-section of the materials were taken.

### 2.4. X-ray Diffraction (XRD)

Structures of CNC, electrospun fibers and PLA nanocomposites were evaluated with X-ray diffraction (XRD). XRD patterns were measured using a Siemens Diffractometer D5000 (Siemens AG, Erlangen, Germany) (30 mA and 40 kV) using CuKa (λ = 1.54 Å) radiation at room temperature. All scans were performed in a 2θ range 2°–12° at 0.02°/s.

### 2.5. Thermal Properties

Thermogravimetric analyses (TGA) of CNC, electrospun fibers and PLA composites were carried out using a Mettler Toledo Gas Controller GC20 Stare System (Schwerzenbach, Switzerland) TGA/DCS. Samples (ca. 9 mg) were heated from 20 to 600 °C at 10 °C min^−1^ under nitrogen atmosphere (flow rate 50 mL min^−1^).

Differential Scanning Calorimetry (DSC) analyses were also performed with a Mettler Toledo DSC-822e calorimeter (Schwerzenbach, Switzerland). Thermograms were obtained from −20 to 220 °C, cooling to −20 °C, and a second heating process to 220 °C with 10 °C min^−1^ heating rate. Sample weight was about 8–10 mg. The degree of crystallinity (*X*_c_) of the PLA materials was deduced using Equation (1):

*X*_c_ = % crystallinity of PLA = 100 × [(Δ*H*_m_ − Δ*H*_cc_)/Δ*H*^0^_m_], (1)

where Δ*H*_m_ is the specific melting enthalpy of the sample (J g^−1^); ΔH_cc_ is the specific cold crystallization enthalpy of the sample (J g^−1^) and Δ*H*^0^_m_ is the specific melting enthalpy of a wholly crystalline PLA (93.1 J g^−1^) [31].

### 2.6. Optical Properties

Films opacity measurements were performed according to the method of Park et al. [32]. Films were cut into rectangular shapes (9 mm × 30 mm) and placed inside the spectrophotometer cell at 600 nm. Five replicates of each film were tested. The opacity of the films were calculated following Equation (2):

(2) $$O=\frac{Abs_{600}}{X}$$

where *O* is the opacity, *Abs*_600_ is the value of absorbance at 600 nm and *X* is the film thickness (mm).

### 2.7. Tensile Testing

Tensile testing of each material was measured using a Zwick Roell model (Ulm, Germany) BDOFB 0.5 TH Tensile Tester, according to ASTM D-882 (American Society for Testing and Materials for Tensile properties of Thin Plastic Sheeting). Strips (10 cm × 2.5 cm) of films were cut using a die cutter and kept at 25 °C and 50% RH (relative humidity) for 48 h before the test. Analyses were carried out with a 1 kN load cell. The initial grip separation was 10 cm and the crosshead speed used was 50 mm min^−1^. Results are the average of 8 specimens for each film.

### 2.8. Oxygen Permeability

The oxygen permeation rates of the PLA materials were determined at 0% RH and 23 °C using an Oxtran model 2/21 ML Mocon (Lippke, Neuwied, Germany). Films were previously purged with nitrogen for a minimum of 16 h in the RH desired, prior to exposure to an oxygen flow of 10 mL/min. Permeation values were determined every 45 min until constant.

### 2.9. Statistical Analysis

A randomized experimental design was considered for the experiments. Data analysis was carried out using Statgraphics Plus 5.1 (StatPoint Inc., Herndon, VA, USA). This software was used to implement variance analysis and Fisher’s LSD (Least Significant Difference) test. Differences were considered significant at *p* < 0.05.

## 3. Results and Discussion

### 3.1. Morphological Results of Nanostructures and Nanocomposites

**Figure 1:**
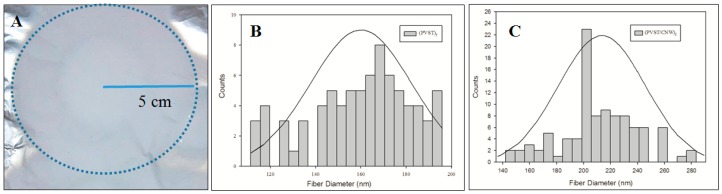
(**A**) macroscopic photograph of (PVST/CNC)_f_ mat; (**B**) histogram of fiber diameter of (PVST)_f_; and (**C**) histogram of fiber diameter of (PVST/CNC)_f_.

Electrospun fibers (PVST/CNC)_f_ were successfully obtained with a final composition of 15.38%, 61.54% and 23.27% (*w*/*w*) of CNC, PV and ST, respectively. Control electrospun fibers had a composition of 72.73% and 27.27% of PV and starch, respectively. As an example, Figure 1A shows the image of the obtained fiber mat electrospun (PVST/CNC)_f_, since, for both fibers, the image was the same. The average sample diameter was 10 cm. As it was already mentioned, the flow rate used was 1.5 mL/h, obtaining a final production rate of 0.165 and 0.195 g/h for fibers (PVST)_f_ and (PVST/CNC)_f_, respectively. The distribution of fiber diameters’ measurements obtained from electrospun fibers (PVST)_f_ and (PVST/CNC)_f_ are presented as histograms in Figure 1B,C, respectively.

**Figure 2:**
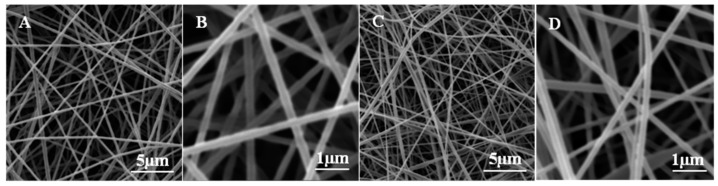
Morphology of electrospun fibers: (**A**) (PVST/CNC)_f_, 10,000×; (**B**) (PVST/CNC)_f_, 40,000×; (**C**) (PVST)_f_, 10,000×; (**D**) (PVST)_f_, 40,000×.

SEM microscopy was a useful tool to observe the morphology of the electrospun fibers (PVST)_f_ and (PVST/CNC)_f_ and the resulting PLA nanocomposites. As Figure 2 shows, it was possible to obtain homogeneous fibers with average diameters of (211.8 ± 18.6) nm and (160.4 ± 18.4) nm for (PVST/CNC)_f_ and (PVST)_f_, respectively. The incorporation of cellulose nanocrystals slightly enhanced the size of resulting fibers. As it was already observed in other studies, CNC could have been aligned along the fiber axis under the electrical field produced during electrospinning process [33,34].

**Figure 3:**
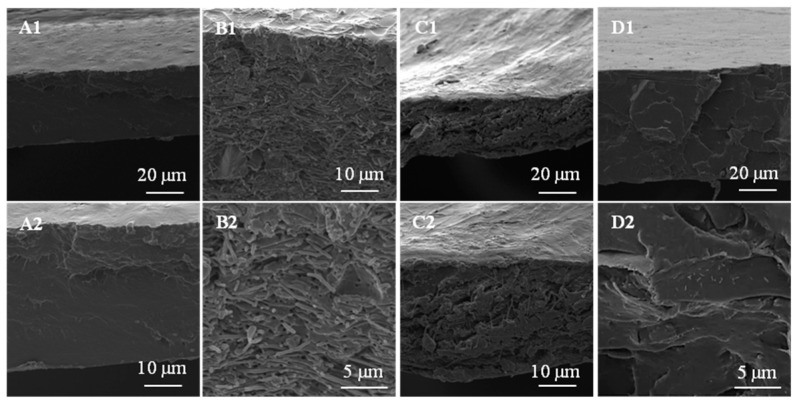
SEM images of PLA composites: (**A**) PLA neat at: (**A1**) 2000×; (**A2**) 4000×; (**B**) 3PLA(PVST/CNC)_f_ at: (**B1**) 4000×; (**B2**) 10,000×; (**C**) 3PLA(PVST)_f_ at (**C1**) 2000×; (**C2**) 4000×; and (**D**) 3PLACNC at (**D1**) 2000×; (**D2**) 10,000×.

Scanning electron microscopy was also used to observe the morphology of developed materials and the distribution of the fibers and CNC into the PLA matrix. Micrographs of material surfaces and cross sections of blank PLA and the materials with maximum concentration of fibers and CNC, as an example, are presented in Figure 3. PLA blank exhibited a smooth surface and apparently a compact and homogeneous structure (Figure 3A1,A2). Micrographs of PLA with 3% of (PVST/CNC)_f_, (Figure 3B1,B2), showed clearly the homogenous distribution of fibers along the thickness of the film, while films with fibers without CNC showed some agglomerations and a heterogeneous fiber distribution (Figure 3C1,C2). Films reinforced with electrospun fibers at low concentration had a smooth surface, but, at higher concentrations, mainly at 3% (*w*/*w*), composites presented a rough surface, although materials with CNC containing fibers were visually more uniform.

Meanwhile, as Figure 3D1,D2 shows, nanocomposites with CNC presented a smooth surface similar to a blank sample, although it was possible to observe certain agglomerations when CNC concentration increased. Thus, micrographs confirmed that the dispersion of these nanofillers was improved when they were embedded in the electrospun fibers.

### 3.2. X-ray Analysis Results

**Figure 4:**
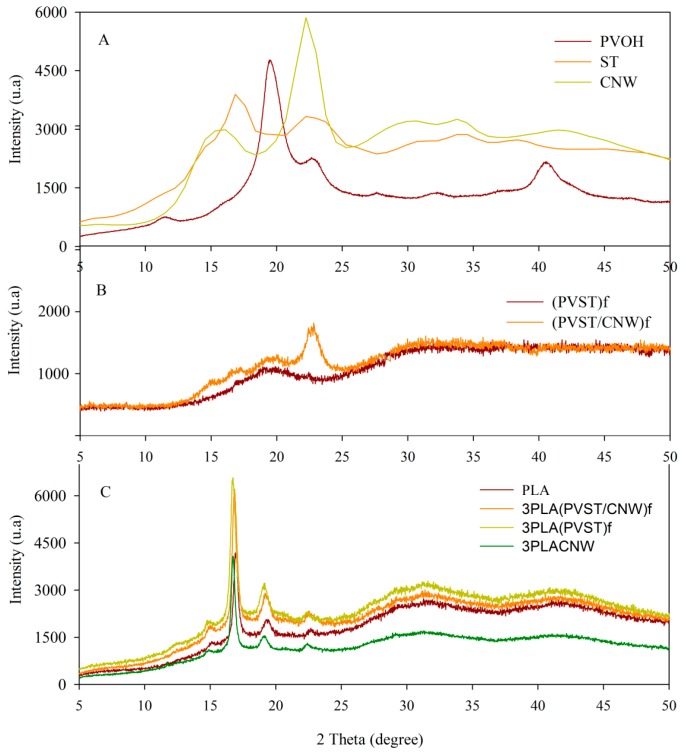
X-ray diffraction patterns of CNC (cellulose nanocrystals), electrospun fibers and PLA based composites.

X-ray diffraction (XRD) patterns of fillers, CNC and electrospun fibers (PVST)_f_ and (PVST/CNC)_f_, and PLA composites at highest concentration of fillers, as an example, are plotted in Figure 4A–C. Diffraction pattern of CNC exhibited a sharp peak at 2θ = 22.34°, corresponding to the crystallographic plane 002, and the cellulose shoulder at 2θ = 15.5°, which is normally assigned to the cellulose I structure [35,36]. PV and starch blank polymers (not processed through electrospinning) were also analyzed, aiming to study the effect of the electrospinning process in the crystallinity of these polymers. As Figure 4A shows, the PV diffraction pattern presented characteristic peaks at 2θ = 11.5°, 19.5°, 22.6°, 32.1° and 40.5° that were attributed to the semi-crystalline nature of the polymer [37,38,39]. The crystal structure of starch can be associated with two crystalline polymorphic forms: A- and B-type, depending on the composition. These crystal structures have been extensively studied and consist of left-handed parallel stranded double helices packed in monoclinic and hexagonal unit cells for the A and B-type crystallites, respectively. Typical A-type-X-ray diffraction patterns presents peaks at 15°, 18° and 23° [40,41]. On the other hand, X-ray studies of electrospun fibers were performed not only to observe the effect of the electrospinning process, but also the incorporation of CNC on PV and starch. As Figure 4B shows, XRD of electrospun fibers (PVST)_f_ presented a considerable loss of crystallinity, showing only a broad shoulder with the maximum around 19.5° corresponding certainly to PV. Previous X-ray diffraction studies have already shown a complete destruction of crystallite integrity as a function of moisture content and temperature. Undoubtedly, however, the main factors that affected crystallinity of both polymers were the interaction between both polymers and the electrospinning process. Rapid processes, such as electrospinning, generally hinder development of crystallinity. Thus, electrospun fibers presented a low degree of crystallinity. Several authors argue that crystallinity decreases as a consequence of the fast solvent evaporation rate that leads to a low molecular arrangement. Furthermore, the starting material for electrospinning is a solution where the polymer has no crystal structure. Due to the very large spinning rate, practically no crystallization occurred. It is, however, possible that the electrospun polymer may undergo some crystallization, as was the case of fibers with CNC that presented a slight enhancement on characteristic peaks from both polymers and clearly a peak corresponding to the presence of CNC at 2θ = 22.3°. As Figure 4B shows, although the electrospinning process reduced the crystallinity of PV and starch, the incorporation of CNC in the fibers implied a rise in the intensities of peaks at 15°, 19.5° and 22.6°. XRD analysis confirmed that CNC showed some nucleating effect. 

X-ray diffraction patterns of developed composites were also performed to obtain information about material crystallinity (Figure 4C). PLA control exhibited characteristics diffraction peaks at a small peak at 2θ = 14.9°, 16.4°, 19.1° and 22.5°, which agreed with data obtained by Pagés et al. [42]. The incorporation of fillers did not affect crystallinity values, and, although this method is not quantitative, it was possible to observe some enhancement on crystallinity degree, which was confirmed by DSC measurements.

### 3.3. Thermal Properties of Nanofillers and Developed Nanocomposites

**Figure 5:**
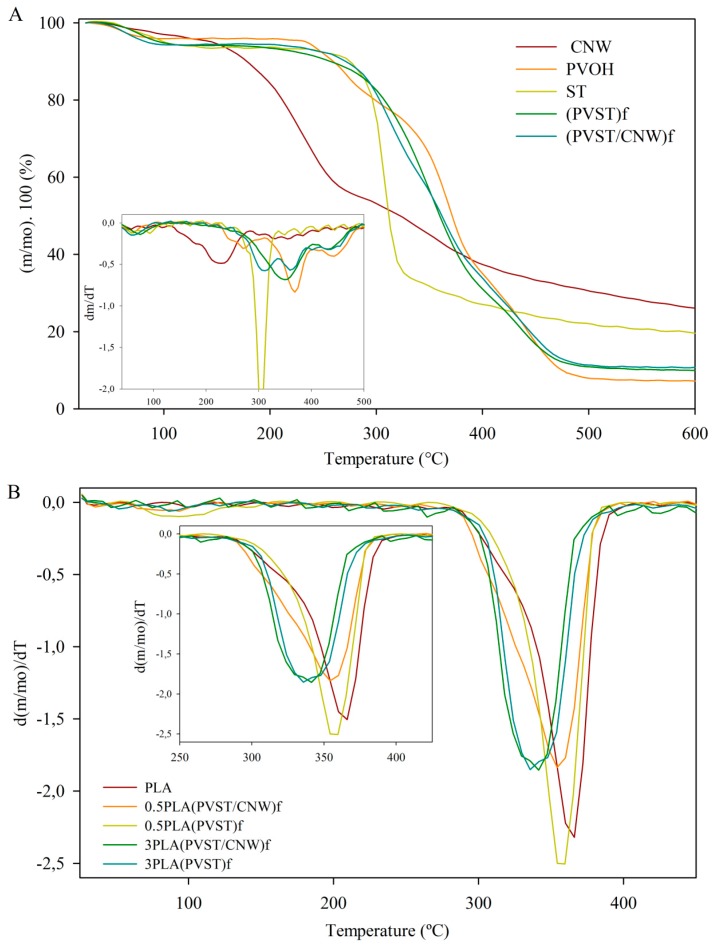
(**A**) TGA (thermogravimetric analysis) curves of individual components. Insert: DTGA (derivative of the TGA curve) of curves; (**B**) DTGA of PLA based composites.

Thermal analyses were performed to study the effect on the thermal properties of: (i) electrospinning process on PV and starch; (ii) the incorporation of CNC; and (iii) the presence of these nanofillers in PLA. Figure 5A shows the mass (%) and the derivative of mass with respect to the temperature (DTGA) of all components used in the development of the electrospun fibers. The initial mass decreasing below 100 °C was attributed to water loss. The thermogram of CNC reported cellulose degradation processes, such as depolymerization, dehydration and decomposition of glycosyl units that occurred at earliest temperatures. The first process corresponded to the degradation of the most accessible regions, which were highly sulphated, and the second process corresponded to the breakdown of the crystalline fraction not attacked by the sulphuric acid, observed in the DTGA curve as a small shoulder [43,44]. A thermogram of starch presented the main peak of degradation at 307 °C, which corresponds to the pyrolytic decomposition phase of amylose and amylopectin. In the case of pure PV, a shoulder was observed followed by a peak between 220 and 415 °C, which were related to the detachment of side groups that forms water, acetic acid and acetaldehyde as by-products. The main peak at 370 °C was associated with thermal degradation of crystalline PV, and the thermal degradation in the molten state occurred as a shoulder of the main peak [45]. Barrera et al. [46] suggested that the detachment of the side groups is the main mechanism for the thermal degradation of PV. The peak observed at 468 °C corresponded to thermal degradation of the PV backbone.

Electrospun fibers (PVST)_f_ presented two degradation processes related to the structural decomposition of starch followed by PVs. Degradation of PV of electrospun fibers was shifted to lower temperatures compared to the pure PV, probably due to the interaction with starch and due to the electrospinning process that caused changes in the polymer structure, as XRD studies suggested previously. Although three degradation processes were observed in the case of (PVST/CNC)_f_, the main degradation process was shifted to higher temperatures when compared to (PVST)_f_, probably because the incorporation of CNC improved its thermal stability. This result indicated that CNC acted as a thermal barrier due to the formation of hydrogen bonds between CNC and polymers, indicating certain compatibility between the components [47].

Figure 5B shows the derivative of the curves of mass loss with the temperature of PLA materials with lowest and highest concentration of fibers, as an example, and Table 2 presents the temperatures of maximum degradation of all composites. The first aspect to be mentioned is that all composites presented one unique degradation process that indicated a good compatibility between the components. Nevertheless, as it is clearly observed in Figure 5B, the incorporation of CNC through electrospun fibers (PVST/CNC)_f_ slightly decreased the thermal stability of composites, and this effect enhanced as the concentration of CNC increased. As control materials with (PVST)_f_ also presented this decrease, it was certainly due to the earlier degradation of electrospun fibers based on PV and starch polymers. The presence of CNC on the fibers also protected the materials from this advancement on degradation, showing its thermal barrier effect already observed.

Table 2 summarizes the significant thermal properties of all PLA composites obtained by DSC during the second heating process. Enthalpies to calculate crystallinity degree were corrected for PLA content. In general, glass transition temperatures were not significantly altered by the incorporation of CNC through electrospun fibers. However, the presence of both fibers, (PVST/CNC)_f_ and (PVST)_f_, favored the cold crystallization process, decreasing significantly the temperature at which crystallization began.

Glass transition temperatures of composites presented values similar to those reported in Lizundia et al. studies for PLA [48]. These values were lower than *T*_g_ obtained in the literature. The reason for the difference could be related to the different obtaining process, the use of plasticizer and the origins of the samples. In addition, the nanocomposites with a low concentration of freeze-dried CNC and embedded CNC into fibers presented a great decrease on *T*_g_. Fortunati et al. studies have already showed this effect in PLA including cellulose nanocrystals and a surfactant for dispersion due to the plasticizing of PLA by desorbed surfactants, or the modification of CNC by lactide oligomers [49,50].

As was already observed by Martinez-Sanz et al. [28], a good dispersion of nanofillers acted as nucleating agent, promoting a faster crystallization of PLA during heating. As Table 2 shows, values of cold crystallization peaks of these composites and control samples with electrospun fibers (PVST)_f_ were reduced compared to the PLA and CNC containing PLA nanocomposites, indicating the nucleating action of electrospun fibers [12]. Furthermore, as Table 2 shows, crystallinity degree of materials was strongly enhanced, and this improvement increased as the concentration of fibers increased. This result has also been observed by Espino-Pérez et al. [12] using CNC chemically modified with *n*-octadecyl-isocyanate. The improvement on CNC dispersion linked to their incorporation through electrospun fibers increased the number of interface/contact filler with the polymer matrix. Other works have named the transcrystalinity effect, the fact that fibers revealed a nucleating role, inducing a robust crystalline morphology of PLA [51]. The presence of freeze-dried CNC also promoted crystallization, but at a lower rate.

### 3.4. Optical Properties

**Figure 6:**
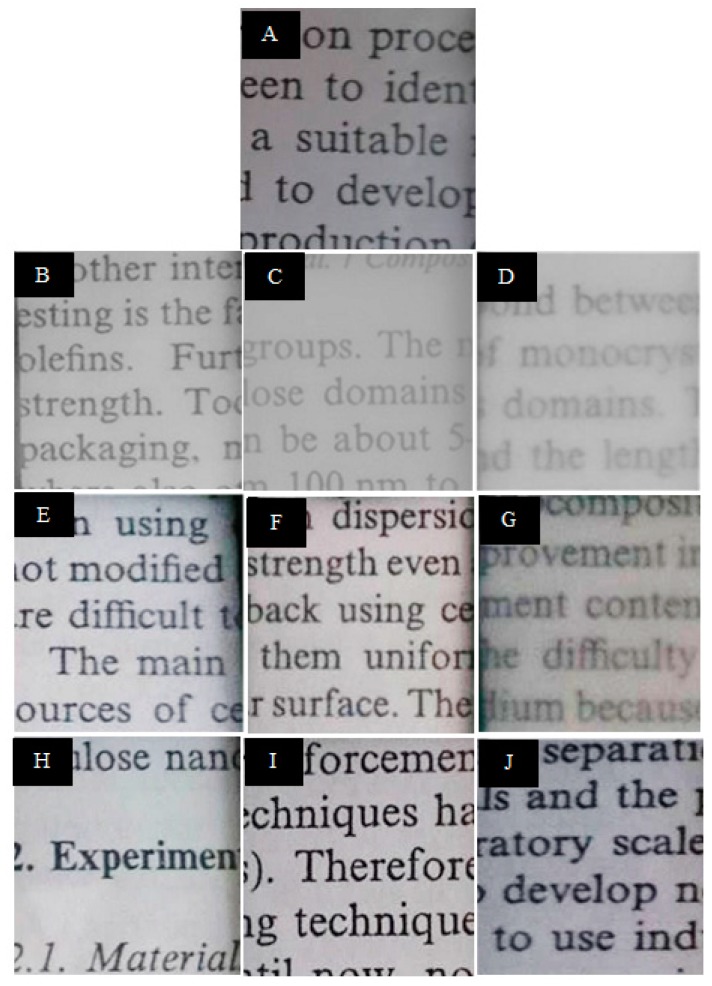
Images of developed PLA materials: (**A**) PLA; (**B**) 0.5PLA(PVST/CNC)_f_; (**C**) 1PLA(PVST/CNC)_f_; (**D**) 3PLA(PVST/CNC)_f_; (**E**) 0.5PLA(PVST)_f_; (**F**) 1PLA(PVST)_f_; (**G**) 3PLA(PVST)_f_; (**H**) 0.5PLACNC; (**I**) 1PLACNC; (**J**) 3PLACNC.

Table 3 shows the opacity of PLA-based films. As Figure 6 shows, the transparency decreased significantly (*p* < 0.05) by increasing electrospun fibers content, and, as it was expected, this effect was higher with the presence of CNC in the fibers. The decrease in transparency as a result of the addition of nanofibers or nanofillers had also been reported before [52]. Although the fillers and polymeric matrix of the composites exhibit excellent translucency, the fillers increased light scattering in the polymer-filler interface and produced opaque materials. The opacity of the composites depends on the sizes of the filler particle. When a particle shrinks to a fraction of the wavelength of visible light (400–800 nm), it will not scatter that particular light. However, if the particle size is far below the wavelength of light, it will not scatter or absorb the light, resulting in the human eye’s inability to detect the particles. This was the case in nanocomposites with freeze-dried CNC at low concentration because opacity increased when concentrations were 1 and 3% due to the appearance of agglomerations.

Since the values of transparency are greatly important in food packaging applications, an option to decrease the opacity would be decreasing the concentration of PV and ST by increasing the concentration of CNC into the fibers or decreasing the diameter of electrospun nanofibers. A surface modification of nanofibers would improve miscibility into the PLA matrix, also diminishing this effect, as it was already seen by Espiro-Pérez et al., whose modified nanocrystals decreased loss of transparency [12].

### 3.5. Mechanical Properties

The mechanical properties of PLA nanocomposites with CNC embedded into electrospun fibers and control materials are summarized in Table 4. The mechanical properties of the films were characterized by Young modulus (YM), tensile strength (TS) and elongation at break (EB) values. In general, a high variance of the results linked to the heterogenous character of the samples was observed. This fact is common when samples are obtained through casting. Results reflected the influence of the electrospun fibers based on two additional polymers and CNC on the stiffness, tensile strength and elongation at break compared with PLA control and PLA nanocomposites with freeze-dried CNC. As Table 4 shows, the addition of electrospun fibers with and without CNC had a major effect on the YM whose values were strongly reduced, and TS values were only decreased at the highest concentration of fibers. Interestingly, composites with electrospun fibers at 1% showed slightly higher values of YM and TS than materials with highest concentration. EB results were in agreement with this effect. At high concentrations, the fibers behaved more as plasticizer than as reinforcement. The incorporation of PV and starch soften the material, obtaining materials more flexible and with higher ductility degree. Probably, the electrospun fibers, with approximately 200 nm diameter, were able to be localized between PLA polymeric chains, resulting in films more flexible and less fragile. Martinez et al. studied the incorporation of bacterial cellulose nanocrystals into PLA through PLA electrospun fibers, and their results have also shown that the electrospinning preincorporation method led to nanocomposites with greater elongation at break [28]. 

Although previous works have shown that the presence of CNC resulted in more rigid materials, in this case, PLA nanocomposites with freeze-dried CNC did not present enhancement of YM, probably due to non-homogeneous dispersion of nanocrystals. Previous works that reported an increase in YM and TS normally were accompanied by great reduction of EB values [16,28]. 

Elongation at break results were not significantly altered, only in the case of PLA nanocomposites with freeze-dried CNC, whose values were greatly decreased. Mechanical properties are very sensitive to interfacial adhesion in a composite, and, in general, elongation at break decreased when interfacial affinity was not ensured. The agglomeration of CNC resulted in break points of the materials. Thus, the incorporation of CNC through fibers protected materials from this effect. These results are positive, since the incorporation of nanocrystals normally tend to diminish these values [53,54]. The fact that the improvement on elongation at break values was due to the presence of PV and ST is clear, since 3PLA(PVST)_f_ was the material that presented approximately an increase in EB of 38%. On the other hand, the rise in resistance due to the increase of crystallinity and presence of CNC was disturbed by the structural characteristics of PV and ST. Results indicated that the incorporation of electrospun fibers of PV and starch generated a flexible but less resistant material. In general, the presence of embedded CNC into the electrospun fibers with their reinforcing nature attempted to diminish the plasticizing effect provided by PV and starch polymeric fibers, although some case differences were not significant. Other works have called this effect the reinforcing plasticizing phenomenon. Although the addition of reinforcing agents, which act as stress concentrating components, in polymeric materials, may typically result in a reduction of the elongation at break, when strong interactions, such as hydrogen bonding, take place between the matrix and the nanofiller, the stress concentration effect is prevented to a certain extent [28,55].

### 3.6. Oxygen Permeability

Table 3 demonstrates the oxygen permeability of developed PLA films. Although films were obtained by casting, the oxygen permeability value of the blank sample was comparable to literature values, approximately 2.3. e^−18^ m^3^ m/m^2^ s Pa [12,28,56,57,58]. Results demonstrated the incorporation of cellulose nanocrystals improved oxygen barrier properties of nanocomposites. Contrary to other works, the incorporation of freeze-dried CNC only showed a slight improvement in the oxygen barrier, most probably due to poor interfacial adhesion and aggregate presence [16,28]. On the other hand, when CNC were incorporated by embedding them into electrospun fibers, oxygen permeability values were greatly reduced, mainly at the highest concentration where reduction achieved about 60%.

Transport properties are known to be strongly influenced by tortuous path altering factors including shape and aspect ratio of the filler, degree of dispersion and filler loading [6,10,15]. Thus, when nanofillers had better dispersion and adhesion to the matrix, a higher barrier property was shown (see Figure S1). This fact was also in accordance with thermal data related to crystallinity rise due to fiber-induced nucleation since nanocomposite 3PLA(PVST/CNC)_f_ was the composite that exhibited highest crystallinity index and oxygen barrier. The advantage to increase flexibility without being affected by barrier properties is an important achievement in this work. In most cases, in order to improve the mechanical properties, in particular flexibility, PLA plasticization is required. However, the use of plasticizers generally induces a decrease in the barrier properties [42].

Finally, composites with (PVST)_f_ also presented an important reduction of oxygen permeability values, revealing that the presence of these fibers was more effective than the incorporation of CNC. This is in agreement with low permeability values that present these polymers at low relative humidity. 

## 4. Conclusions

The main goal of this study was to improve the dispersion of cellulose nanocrystals (CNC) within PLA matrices in order to improve physical properties. The strategy used was the incorporation of CNC into electrospun fibers of polyvinyl alcohol (PV) and starch (ST), (PVST/CNW)_f_. Electronic microscopy revealed that it was possible to obtain a better dispersion of nanofillers when CNC was incorporated by means of electrospun nanofibers, although transparency of materials was diminished. There were some improvements in the properties of the nanocomposite materials compared to pure PLA and nanocomposites with freeze dried CNC. Results indicated that the incorporation of (PVST/CNC)_f_ modified the structural properties of PLA, and the performance of CNC on the film for reinforcement is better through electrospun nanofibers. PLA crystallinity of materials containing electrospun fibers with and without CNC was enhanced, leading to a decrease in oxygen permeability values. Interestingly, marked decreases were found in samples containing (PVST)_f_ electrospun fibers, indicating that CNC has a marginal effect on the property decrease. Incorporation of CNC embedded into electrospun nanofibers resulted in less stiff and slightly more flexible composites. 

## Figures and Tables

**Table 1:** Composition (%) of developed films.

Film Samples	PLA	(PVST/CNC)_f_	(PVST)_f_	CNC
PLA	100	-	-	-
0.5PLA(PVST/CNC)_f_	96.75	3.25	-	-
0.5PLA(PVST)_f_	97.25	-	2.75	-
0.5PLACNC	99.5	-	-	0.5
1PLA(PVST/CNC)_f_	93.5	6.5	-	-
1PLA(PVST)_f_	94.5	-	5.5	-
1PLACNC	99	-	-	1.0
3PLA(PVST/CNC)_f_	80.5	19.5	-	-
3PLA(PVST)_f_	83.5	-	16.5	-
3PLACNC	97	-	-	3.0

Abbreviations: PV: polyvinyl alcohol; ST: starch; PLA: poly(acid lactic); CNC: cellulose nanocrystal; (PVST)f: electrospun nanofibers; (PVST/CNC)f: electrospun nanofibers containing CNC.

**Table 2:** Thermal properties of poly(lactic acid) PLA-based developed films.

Films	*T*_deg_	*T*_g_ (°C)	*T*_cc_ (°C)	Δ*H*_cc_ (J/g)	*T*_m_ (°C)	Δ*H*_m_ (J/g)	*X*_c_′ (%)
PLA	365.6 ± 2.6 ^e^	39.0 ± 1.4 ^b,c^	90.6 ± 0.3 ^d^	25.7 ± 0.2 ^c,d,e^	148.2 ± 0.8 ^c,d^	30.3 ± 0.5 ^c,d,e^	3.2 ± 1.4 ^a,b^
0.5PLA(PVST/CNC)_f_	356.7 ± 2.6 ^b,c,d^	34.1 ± 0.2 ^a^	84.6 ± 0.7 ^a,b^	21.8 ± 1.4 ^b^	145.7 ± 0.2 ^b^	28.1 ± 2.1 ^b,c^	6.8 ± 0.7 ^d^
0.5PLA(PVST)_f_	356.4 ± 3.2 ^b,c,d^	43.8 ± 0.7 ^d^	94.9 ± 1.1 ^f^	20.6 ± 0.3 ^a^	149.8 ± 0.2 ^e^	23.1 ± 0.9 ^a^	2.7 ± 0.7 ^a^
0.5PLACNC	347.3 ± 15.6 ^a,b^	35.4 ± 0.4 ^a^	85.4 ± 0.5 ^b^	24.6 ± 1.1 ^c^	146.1 ± 0.6 ^b^	28.9 ± 1.2 ^b,c,d^	4.7 ± 0.1 ^b,c^
1PLA(PVST/CNC)_f_	356.7 ± 3.9 ^b,c,d^	39.6 ± 0.1 ^b,c^	88.7 ± 1.7 ^c^	22.7 ± 0.5 ^b^	147.0 ± 0.5 ^b,c^	26.9 ± 0.4 ^b^	4.5 ± 0.1 ^b^
1PLA(PVST)_f_	348.1 ± 6.0 ^a,b^	39.1 ± 1.0 ^b,c^	89.3 ± 0.6 ^c,d^	25.1 ± 0.1 ^c,d^	148.2 ± 0.1 ^c,d^	29.2 ± 0.1 ^b,c,d,e^	4.4 ± 0.2 ^a,b^
1PLACNC	362.5 ± 1.5 ^c,d^	38.4 ± 3.7 ^c^	95.2 ± 1.6 ^f^	25.4 ± 2.6 ^d,e^	148.6 ± 0.2 ^d,e^	26.9 ± 0.7 ^b^	3.6 ± 0.7 ^a,b^
3PLA(PVST/CNC)_f_	353.4 ± 10.4 ^b,c^	39.4 ± 0.7 ^c^	82.1 ± 1.8 ^a^	24.9 ± 1.2 ^c,d,e^	144.6 ± 0.2 ^a^	31.0 ± 0.4 ^d,e,f^	6.5 ± 1.7 ^d^
3PLA(PVST)_f_	339.3 ± 3.6 ^a^	39.8 ± 2.0 ^c^	83.4 ± 2.2 ^a^	25.8 ± 2.3 ^e^	143.6 ± 2.2 ^a^	31.7 ± 3.8 ^e,f^	6.4 ± 1.6 ^c,d^
3PLACNC	363.1 ± 1.2 ^c,d^	38.5 ± 0.8 ^b^	92.5 ± 0.8 ^e^	29.6 ± 0.4 ^f^	149.7 ± 0.4 ^e^	32.6 ± 0.2 ^f^	4.6 ± 1.1 ^b^

Lower case letters ^a–e^ indicate significant differences in a thermal parameter among the materials. (*T*_g_: glass transition temperature; *T*_cc_ and *T*_m_: cold crystallization and melting temperatures, respectively; *ΔH*_cc_ and *ΔH*_m_: cold crystallization and melting enthalpies, respectively; *X*_c_′: crystallinity degree).

**Table 3:** Opacity values and oxygen permeation results for PLA based materials.

Films	Opacity Index	P O_2_ (m^3^ m/m^2^ s Pa)
PLA	2.2 ± 0.3 ^a^	2.29. e^−18^
0.5PLA(PVST/CNC)_f_	6.8 ± 0.8 ^a,b^	7.45. e^−18^
0.5PLA(PVST)_f_	5.4 ± 0.8 ^a,b^	1.52. e^−18^
0.5PLACNC	6.2 ± 1.4 ^a,b^	1.71. e^−18^
1PLA(PVST/CNC)_f_	6.4 ± 0.4 ^a,b^	1.52. e^−18^
1PLA(PVST)_f_	7.6 ± 1.2 ^a,b^	6.83. e^−19^
1PLACNC	7.1 ± 2.1 ^a,b^	1.83. e^−18^
3PLA(PVST/CNC)_f_	23.9 ± 8.6 ^d^	9.57. e^−19^
3PLA(PVST)_f_	14.6 ± 4.2 ^c^	1.53. e^−18^
3PLACNC	9.0 ± 3.5 ^b,c^	1.80. e^−18^

Lower case letters ^a–c^ indicate significant differences in opacity among the materials.

**Table 4:** Mechanical properties of developed PLA composites.

Material	Young’s Modulus	Tensile Strength	Elongation at
	(MPa)	(MPa)	Break (%)
PLA	1607.1 ± 164.2 ^c^	47.9 ± 4.6 ^c,d^	3.4 ± 0.4 ^a,b^
0.5PLA(PVST/CNC)_f_	1170.3 ± 207.3 ^a,b^	47.7 ± 2.6 ^c^	3.9 ± 0.3 ^d^
0.5PLA(PVST)_f_	1116.5 ± 248.1 ^a,b^	43.7 ± 4.9 ^b,c^	4.1 ± 0.3 ^d,e^
0.5PLACNC	1608.9 ± 132.3 ^c^	40.3 ± 2.2 ^b^	2.8 ± 0.4 ^a,b^
1PLA(PVST/CNC)_f_	1342.5 ± 249.8 ^b,c^	46.9 ± 3.5 ^c,d^	3.5 ± 0.3 ^b,c,d^
1PLA(PVST)_f_	1306.7 ± 73.2 ^b,c^	43.9 ± 2.5 ^b,c^	3.7 ± 0.3 ^c,d^
1PLACNC	1123.8 ± 247.3 ^a,b^	41.9 ± 4.2 ^b,c^	2.9 ± 0.6 ^a,b^
3PLA(PVST/CNC)_f_	858.2 ± 114.7 ^a^	32.4 ± 2.1 ^a^	3.8 ± 0.3 ^d^
3PLA(PVST)_f_	860.1 ± 161.6 ^a^	29.0 ± 2.4 ^a^	4.7 ± 0.5 ^e^
3PLACNC	1239.9 ± 220.3 ^b^	31.2 ± 3.3 ^a^	2.6 ± 0.3 ^a^

Lower case letters ^a–d^ indicate significant differences in a mechanical parameter among the materials.

## Supplementary Materials

The following are available online at www.mdpi.com/2073-4360/9/4/117/s1, Figure S1: Schematical representation of the tortuous diffusion path generated by CNC containing fibers (PVST/CNC)_f_ into PLA materials.
