# Supplementary material for: Novel Polyvinyl Alcohol/Starch Electrospun Fibers as a Strategy to Disperse Cellulose Nanocrystals into Poly(lactic acid)
Source: Polymers (Basel). 2017 Apr 7;9(4):117. doi: 10.3390/polym9040117 (PMC6432082; doi:10.3390/polym9040117)
Supplement: Supplementary file 1 [file polymers-09-00117-s001.pdf]

Supplementary Materials: Novel Polyvinyl Alcohol/Starch Electrospun Fibers as a Strategy to Disperse Cellulose Nanocrystals into Poly(lactic acid)

Carol López de Dicastillo, Karina Roa, Luan Garrido, Alejandro Pereira and Maria Jose Galotto

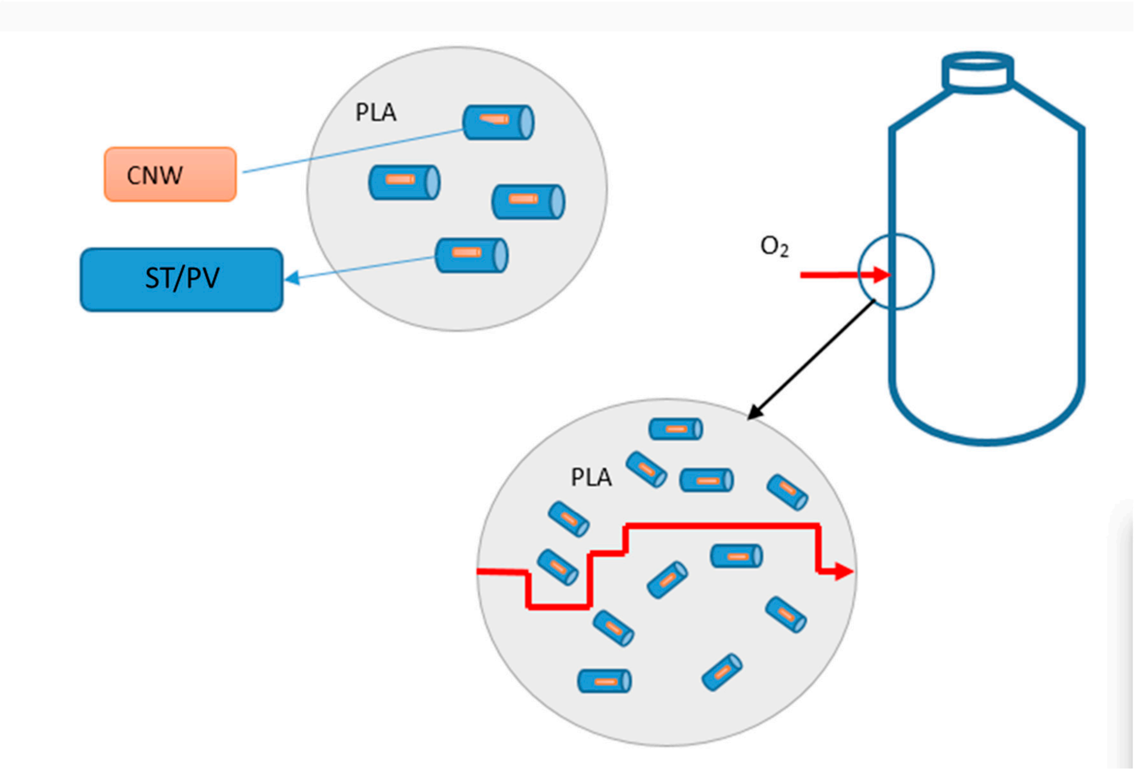

Figure S1. Schematical representation of the tortuous diffusion path generated by CNC containing fibers (PVST/CNC)f into PLA materials.
